# Nuclear Ribosomal ITS1–5.8S rDNA-ITS2 Region as a Phylogenetic Marker for *Selenicereus grandiflorus* and Related Species (Cactaceae: Cactoideae: Phyllocacteae)

**DOI:** 10.3390/ijms27167166

**Published:** 2026-08-11

**Authors:** Alexander V. Rodionov, Edward M. Machs, Evgenia L. Romanova, Alexander G. Dyomin, Ivan N. Shulzhenko, Alexander A. Gnutikov, Nikolai N. Nosov, Victoria S. Shneyer, Yuri G. Kalugin

**Affiliations:** 1 Komarov Botanical Institute of the Russian Academy of Sciences, 197376 St. Petersburg, Russia; avrodionov@binran.ru (A.V.R.); emachs@binran.ru (E.M.M.); eromanova@binran.ru (E.L.R.); adyomin@binran.ru (A.G.D.); ishulzhenko@binran.ru (I.N.S.); a.gnutikov@vir.nw.ru (A.A.G.); nnosov@binran.ru (N.N.N.); shneyer@binran.ru (V.S.S.); 2 Faculty of Biology, St. Petersburg State University, 199034 St. Petersburg, Russia; 3 Biological Station Rybachy, Zoological Institute of the Russian Academy of Sciences, 238535 Rybachy, Russia; 4 N.I. Vavilov All-Russian Institute of Plant Genetic Resources (VIR), 190000 St. Petersburg, Russia

**Keywords:** 35S rDNA, Cactaceae, Caryophyllales, 5.8S rRNA pseudogenes, intragenomic rDNA polymorphism, DNA barcoding, molecular phylogeny

## Abstract

The nuclear ITS1–5.8S rDNA–ITS2 region is among the most widely used markers for phylogenetic studies in plants. We sequenced the ITS1–5.8S rDNA–ITS2 region in several species of *Selenicereus*, two species of *Deamia* and *Epiphyllum chrysocardium*. The newly generated sequences were found to be more closely related to ITS and 5.8S rDNA sequences from representatives of other cactus tribes than to the *Selenicereus* ITS sequences previously deposited in GenBank by research group O. Plume. Importantly, all these sequences retained three conserved motifs of the 5.8S rRNA gene that are commonly used to distinguish functional copies from pseudogenes. Then we analyzed available genome assemblies representing 12 species of Cactaceae. In contrast to the ribotypes identified in this study, the previously reported 5.8S rDNA variants in the assembled genomes of *Selenicereus* and *Carnegiea* were represented by a single copy flanked by highly degraded 18S and 28S rDNA sequences. Phylogenetic analyses based on the newly generated ITS sequences and Genbank data supported the monophyly of the subtribes Hylocereinae, Rhipsalideae, and Trichocereinae, as well as the tribes Rhipsalideae, Notocacteae, and Cylindropuntieae. These results demonstrate that the functional ITS1-5.8S rDNA–ITS2 region represents an informative marker for phylogenetic studies within the Cactaceae.

## 1. Introduction

The family Cactaceae Juss. is a monophyletic lineage within the order Caryophyllales and represents one of the most morphologically distinctive groups of flowering plants [1,2,3,4]. The ancestral group of Cactaceae is estimated to have originated during the Late Eocene, approximately 28–35 million years ago [2,5]. Nevertheless, the remarkable morphological and ecological diversity observed in extant cacti is largely the result of a more recent radiation that occurred during the last 5–12 million years [2]. According to current taxonomic estimates, the family comprises at least 1850 species distributed throughout the Americas, with a few species becoming naturalized elsewhere [3].

**Figure 1:**
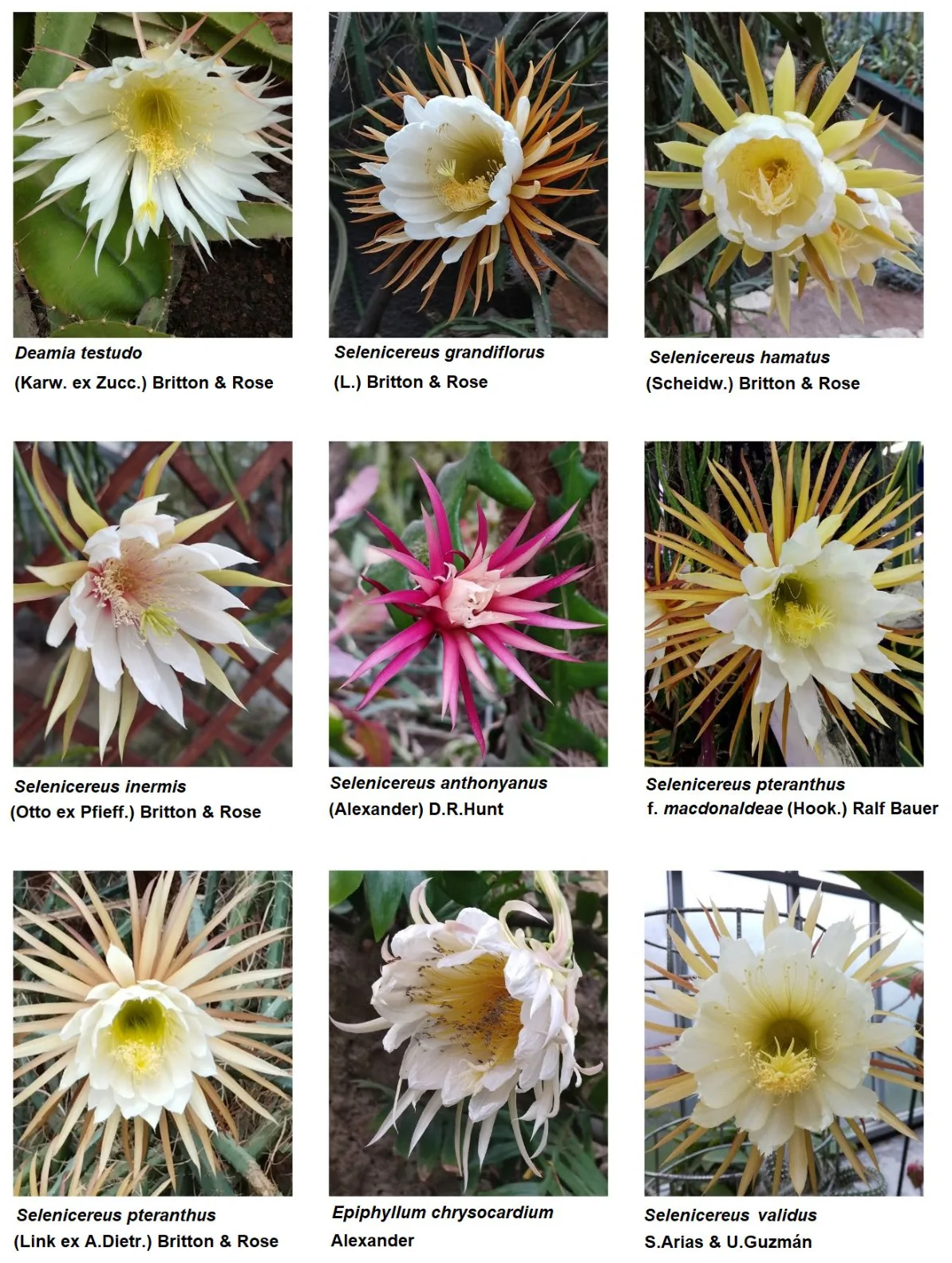
*Selenicereus grandiflorus* and allied species maintained in the living collection of the Komarov Botanical Institute.

Depending on the taxonomic treatment adopted, cactus species are classified into either four [1,2,6] or six [7] subfamilies based on a combination of morphological and molecular phylogenetic evidence. The present study focuses on species closely related to *Selenicereus grandiflorus* (L.) Britton & Rose, a member of the subtribe Hylocereinae Britton & Rose within the tribe Phyllocacteae Salm-Dyck and the subfamily Cactoideae Eaton [1,7]. Owing to its spectacular nocturnal flowers, *S. grandiflorus* is widely cultivated in botanical gardens and private collections and is commonly known as “Queen of the Night”, “Night-blooming Cereus”, “Large-flowered Cactus”, and “Reina de la Noche” [8,9] (Figure 1).

In addition, several economically important species, including *Selenicereus undatus* (Haw.) D.R. Hunt, *S. megalanthus* (K. Schum. ex Vaupel) Moran, and *S. monacanthus* (Lem.) D.R. Hunt, are cultivated worldwide for the production of dragon fruit (pitahaya) [10,11].

Previous phylogenetic studies based on chloroplast markers [12,13,14,15], complete plastome sequences [16], and hundreds of low-copy nuclear loci [7] consistently recovered Hylocereinae as a monophyletic lineage. In these analyses, *Selenicereus* and its allies form a well-supported clade that is sister to Echinocereinae, indicating a stable phylogenetic placement within Phyllocacteae.

The nuclear ribosomal ITS1–5.8S rDNA–ITS2 region is among the most frequently employed molecular markers in plant systematics, DNA barcoding, and phylogenetic reconstruction [17,18,19]. Owing to its relatively rapid evolutionary rate and generally efficient concerted evolution within ribosomal DNA arrays, the ITS region often provides sufficient variation for species-level discrimination while retaining phylogenetic signal at higher taxonomic levels [17,18,19].

During the initial stage of this study, we examined ITS1–5.8S rDNA–ITS2 sequences of *Selenicereus* sensu lato available in GenBank. At that time, the database contained 254 accessions generated by Plume et al. [14] using the custom primer pair ITSC1-F/ITSC1-R. Unexpectedly, these sequences displayed exceptionally high levels of sequence variation. Pairwise comparisons revealed p-distances ranging from 0.16% to 8.0% (excluding indels), with a median value of 4.8%, considerably exceeding the levels between different genera typically reported for plant ITS sequences [19]. Even more strikingly, these sequences differed from ITS sequences of other cactus genera by 22–28%, whereas ITS sequences from most other genera within Cactaceae generally differ by only 3–5% [20,21,22,23,24,25,26,27,28,29].

An additional source of concern was the observation by Plume et al. [14] that all analyzed *Hylocereus* and *Selenicereus* sequences contained substitutions within three highly conserved Viridiplantae motifs of the 5.8S rRNA gene: GAATTGCAGAAWYC, TTTGAAYGCA, and CGATGAAGAACGYAGC. Harpke and Peterson [30] proposed these motifs as diagnostic features for distinguishing functional ITS regions from pseudogenic copies. Consequently, Plume et al. [14] suggested that at least some of the amplified sequences might represent pseudogenes rather than functional ribosomal DNA variants.

These observations prompted us to re-examine the ITS1–5.8S rDNA–ITS2 region in *Selenicereus* and related taxa using the widely employed primer combination ITS1P [31] and ITS4 [32]. In addition to multiple *Selenicereus* species, we analyzed representatives of *Deamia* and *Epiphyllum chrysocardium*. The resulting sequences retained all conserved 5.8S rRNA motifs and showed substantially greater similarity to ITS sequences from other cactus lineages than to the previously published Hylocereinae sequences. Furthermore, phylogenetic analyses based on the newly generated ITS data provided additional insights into the evolutionary relationships among *Selenicereus* and other members of the family Cactaceae. Taxonomic names and ranks generally follow the phylogenomic classification of de Vos et al. [7], except where historical circumscriptions are discussed for comparison with previous studies.

## 2. Results

### 2.1. 5.8S rDNA Genes and Pseudogenes

Comparative analysis of 5.8S rDNA sequences showed that species of *Selenicereus*, as well as two species of *Deamia* and *Epiphyllum*, possess identical 5.8S rDNA sequences. Only one specimen of *S. pteranthus* f. *macdonaldieae* (Hook.) Ralf Bauer (sample 56) exhibited two substitutions, G→C and C→T, corresponding to positions 395 and 397 of the reference sequence of *Mammillaria beneckei* Ehrenb. AM157759 [22]. The 5.8S rDNA sequences generated in this study did not differ from sequences belonging to species of other tribes within the Cactaceae, or in some cases differed by only 1–2 nucleotides. Exceptions were the 5.8S rDNA sequences of *Cephalocereus senilis* (Haw.) K.Schum. AY181585, *Carnegiea gigantea* (Engelm.) Britton & Rose AY181576, and *Escontria chiotilla* (F.A.C. Weber ex K. Schum.) Rose AY181576 [21], which differed from the consensus 5.8S rDNA sequence by 10–18 single nucleotide polymorphisms (SNPs) and insertions/deletions (p-distance 0.06–0.12). These three sequences/loci represented pseudogenic 5.8S rDNA copies containing defects in two of the three conserved motifs identified by Harpke and Peterson [30].

**Figure 2:**
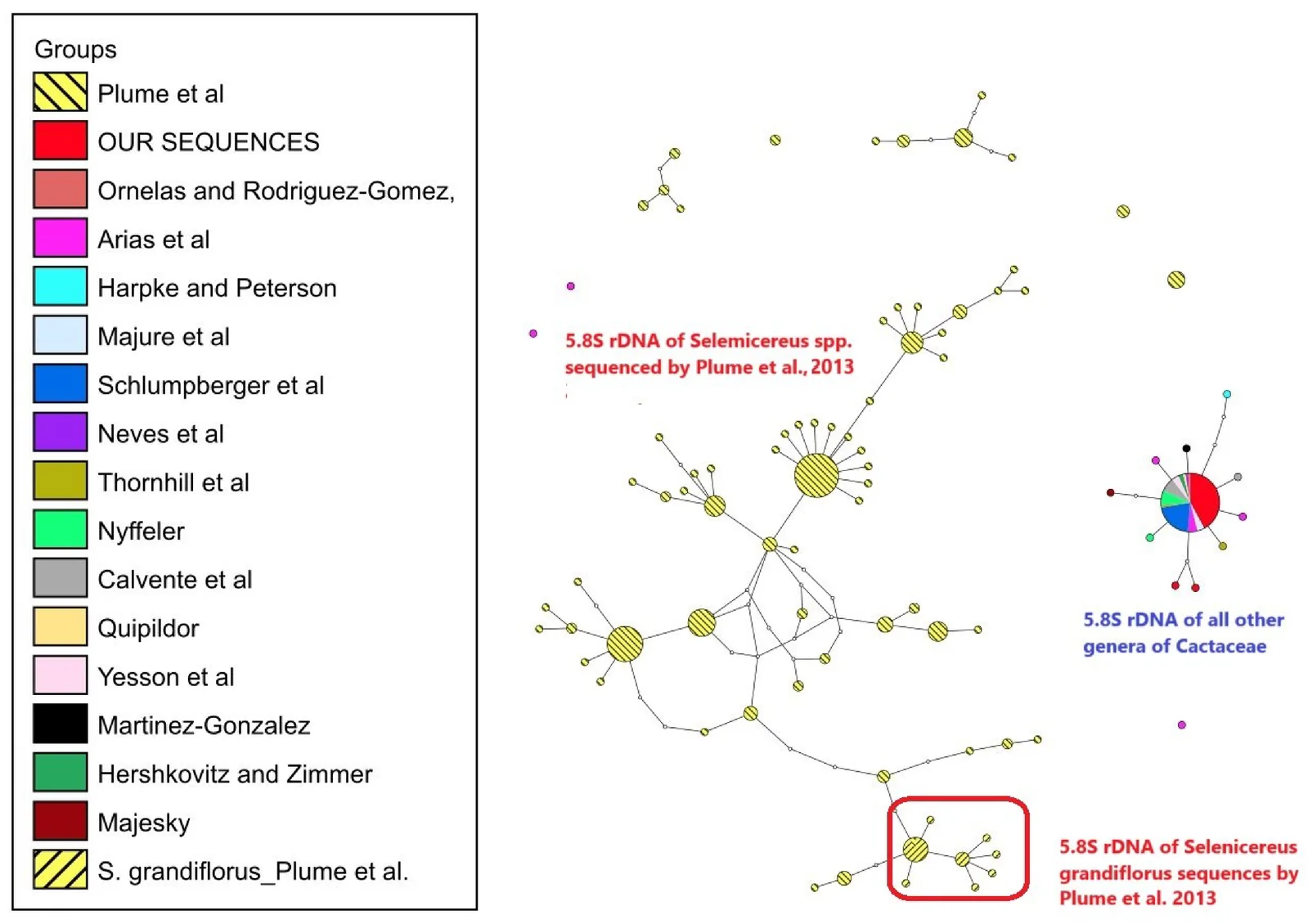
Ribotype network (5.8S rDNA) of representative species from various Cactaceae genera, sequenced across different laboratories and processed using tcsBU. Data source: GenBank. The absolute number of mutational steps (single nucleotide polymorphisms, SNPs) required to parsimoniously connect adjacent ribotypes is indicated along the branches. Circle sizes correspond to the relative frequency of each ribotype within the analyzed sample. A complete list of the sequences used is provided in Table S1. Cited sources: [14,20,21,23,24,25,26,27,29,30,36,37].

To visualize sequence polymorphism in 5.8S rDNA among different genera and tribes of the Cactaceae, we employed the algorithm developed by Templeton et al. [33], which detects ribotype variation using a maximum parsimony approach. Because 5.8S rDNA sequences are highly conserved, we selected one representative species from each cactus genus for which 5.8S rDNA sequences were available in GenBank. Calculations were performed using TCS software [34], and the results were visualized with tcsBU [35]. TCS estimates the probability of association among all ribotypes and indicates the number of mutational steps separating them [33,34]. The resulting network is shown in Figure 2.

The 5.8S ribotypes clearly form two distinct groups: ribotypes sequenced by Plume and co-authors [14] and ribotypes analyzed in other laboratories. All 5.8S rDNA sequences generated in the present study for *Selenicereus* spp., as well as the 5.8S rDNA sequences of *Epiphyllum chrysocardium*, *Deamia testudo*, and *D. chontalensis*, belong to the second group (Figure 2, multicolored circle) and differ markedly from those reported by Plume et al. [14]. The latter forms a separate heterogeneous ribotype network (yellow circles).

**Figure 3:**
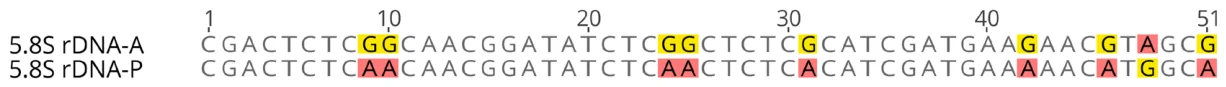
Conserved motifs of the functional 5.8S rDNA-A (highlighted in yellow) and pseudogenic 5.8S rDNA-P (highlighted in red) lineages utilized as diagnostic indicators for functional genes and pseudogenes within cactus genomes.

To investigate the chromosomal localization of the 5.8S rRNA genes represented by the ribotypes identified in this study and by ribotypes reported by Plume et al. [14], we analyzed the available genome assemblies of 12 fully sequenced members of the family Cactaceae. Using BLAST, we searched these assemblies for a conserved fragment of the 5.8S rDNA sequence from the ribotypes identified in our study and for a conserved fragment of the 5.8S rDNA sequence corresponding to the ribotypes reported by Plume et al. [14], hereafter designated 5.8S rDNA-P, whereas the ribotype identified in the present study is designated 5.8S rDNA-A (Figure 3).

The 5.8S rDNA-A sequence was detected in 10 of the 11 analyzed scaffold-level and whole-genome shotgun (WGS) assemblies. The sole exception was the genome assembly of *Cereus fernambucensis* Lem. (GCA_024363205.1), which contained only several short WGS contigs harboring sequences similar, but not identical, to the 5.8S rDNA-A ribotype. Future improvements of the *C. fernambucensis* assembly will likely reveal the canonical 5.8S rDNA-A ribotype as well.

In all assemblies where rDNA clusters were represented by multiple copies (*Carnegiea gigantea* JAKOGI010000148.1, *Cereus jamacaru* DC. CM120792.1, *Lophophora williamsii* (Lem. ex J.F.Cels) J.M.Coult. CAXLBD010019528.1 and CAXLBD010004043.1, *Opuntia basilaris* Engelm. & J.M.Bigelow JBFLFP010000011.1, and *Selenicereus monacanthus* CM104744.1), the 5.8S rDNA-A ribotype was the predominant form and occurred within tandem repeat arrays, which we interpret as nucleolar organizer regions (NORs). This observation strongly suggests that the 5.8S rDNA-A ribotype represents the canonical form of 5.8S rDNA in the family Cactaceae. In chromosome-level assemblies, such tandem arrays were identified on chromosome 10 of *Cereus jamacaru* (CM120792.1) and chromosome 11 of *Selenicereus monacanthus* (CM104744.1).

**Figure 4:**
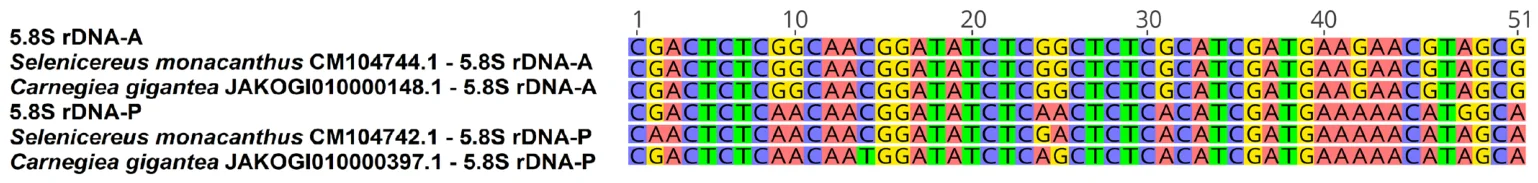
Chromosomal distribution of genomic sequences sharing similarity with the 5.8S rDNA-A and 5.8S rDNA-P ribotypes within the assembled genomes of *Selenicereus monacanthus* and *Carnegiea gigantea*.

Sequences identical to the 5.8S rDNA-P ribotype were not detected either in the assemblies examined above or in the raw-read datasets associated with the corresponding genome projects. The closest relatives of the 5.8S rDNA-P ribotype (94.12% sequence identity) were identified in the chromosome-level assembly of *Selenicereus monacanthus* (CM104742.1, chromosome 9) and in the WGS assembly of *Carnegiea gigantea* (JAKOGI010000397.1) (Figure 4). In both cases, these sequences were present as single-copy (orphan) loci embedded within highly degraded fragments of the 18S and 28S rRNA genes.

**Figure 5:**
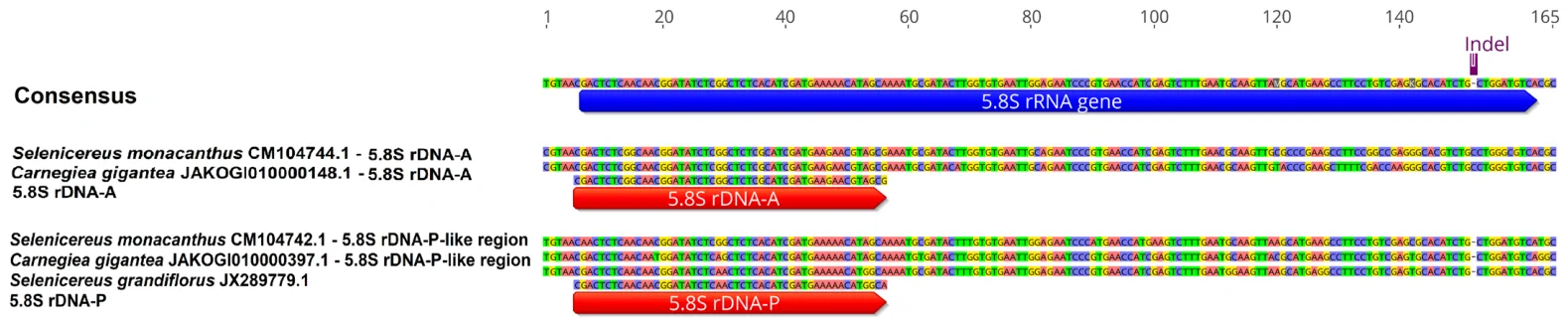
Alignment of the complete canonical 5.8S rDNA-A ribotype sequences with sequences closely related to the 5.8S rDNA-P ribotype in *Selenicereus monacanthus* and *Carnegiea gigantea*, alongside the JX289779.1 sequence of *Selenicereus grandiflorus*.

Alignment of the complete 5.8S rRNA gene sequences from chromosomes 9 and 11 of *Selenicereus monacanthus*, WGS contigs JAKOGI010000148.1 and JAKOGI010000397.1 of *Carnegiea gigantea*, and sequence JX289779.1 of *Selenicereus grandiflorus* annotated by Plume et al. [14] revealed an identical deletion shared by the 5.8S rDNA variants located on chromosome 9 of *S. monacanthus*, contig JAKOGI010000397.1 of *C. gigantea*, and sequence JX289779.1 (Figure 5). This finding indicates that the pseudogene likely originated prior to the divergence of the phylogenetic lineages leading to *Carnegiea* and *Selenicereus*.

**Figure 6:**
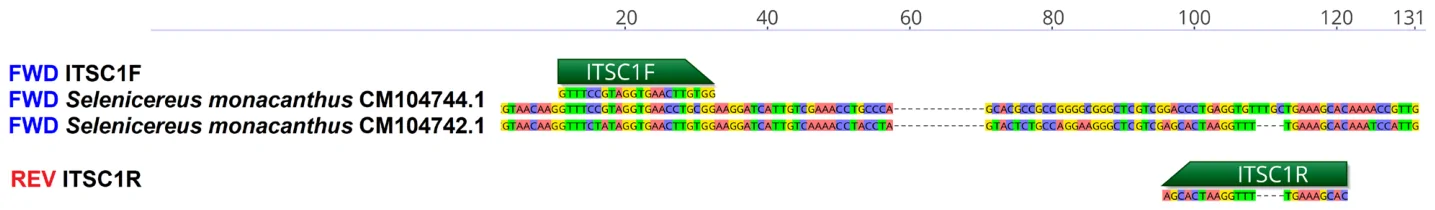
Alignment of the ITSC1-R primer binding site with the 35S rDNA gene sequence of *Selenicereus monacanthus*.

Examination of the primer-binding sites for the ITSC1-F/ITSC1-R primer pair developed by Plume et al. [14] revealed that the corresponding target sequences in the completely sequenced genome of *Selenicereus monacanthus* (GCA_047496255.1) are located on chromosome 9 (assembly CM104742.1). This sequence contains a characteristic four-nucleotide deletion within the ITSC1-R primer-binding sequence that is absent from the canonical rDNA sequence located on chromosome 11 (CM104744.1). Consequently, the ITSC1-F/ITSC1-R primer pair is inherently incapable of amplifying the canonical ITS1–5.8S–ITS2 copy from NOR of *Selenicereus monacanthus* (Figure 6).

**Figure 7:**
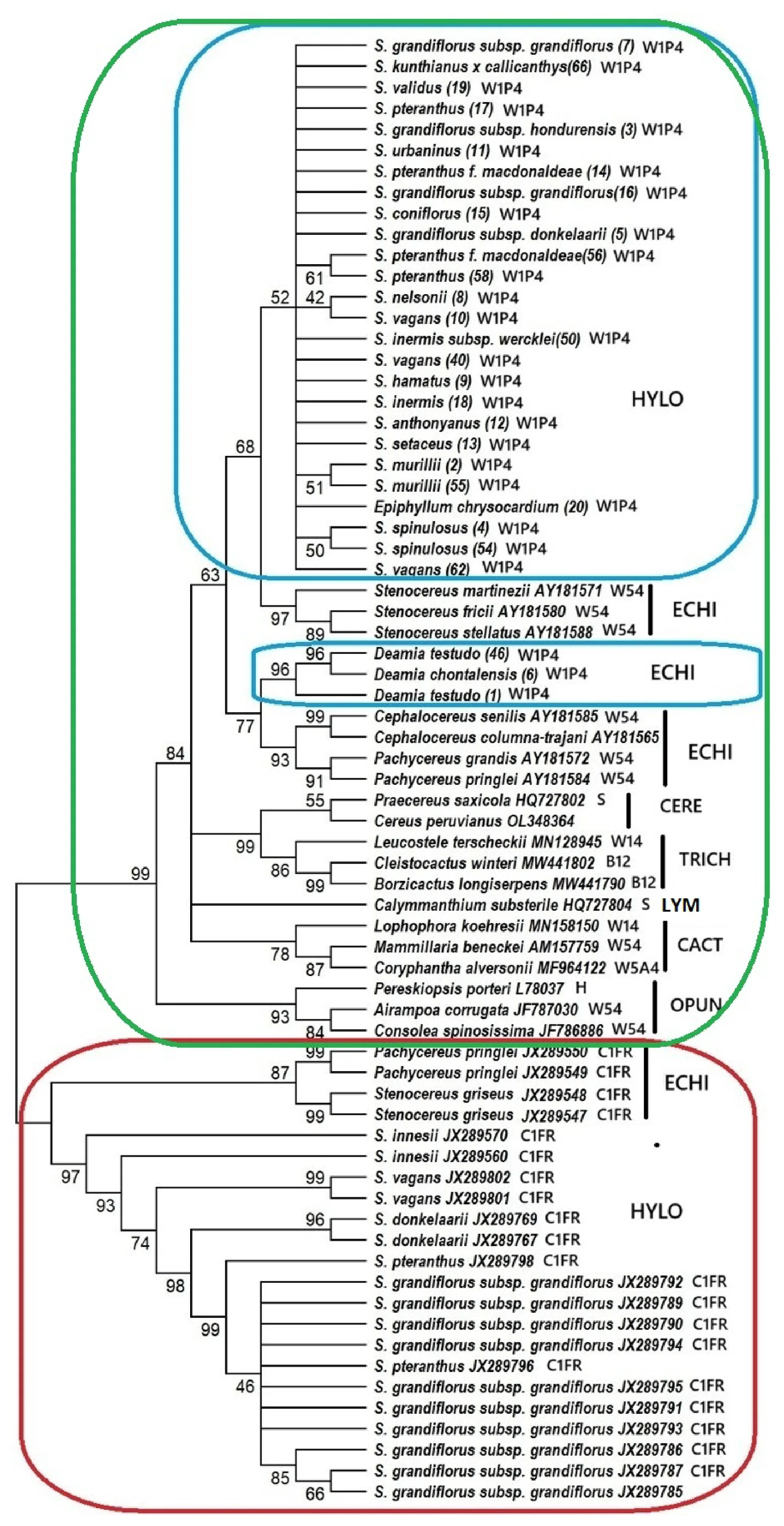
Phylogenetic tree reconstructed via the Minimum Evolution (ME) method based on the sequence alignment of the ITS1–5.8S rDNA-ITS2 region from various Cactaceae species. Numbers in brackets following the species names indicate voucher accessions unique to this study. For sequences retrieved from the GenBank database, the accession numbers are accompanied by the primer pairs used for their amplification: C1FR—the custom primer set developed by Plume et al. [14]; W14 and W54—the standard ITS1+ITS4 and ITS5+ITS4 combinations, respectively, described by White et al. [32]; W1P4—the ITS1P [31] and ITS4 [32] pair; W5A4—the ITS5A [38] and ITS4 [32] combination; H—the 18NRBlr [20] and 26A [39] primer pair; S—the ITS-AF and ITS-AR primers [40]; B54—the 5F and 4R primers [41]; and B12—the diagnostic primers (1) and (2) proposed by von Balthazar et al. [42]. The specific primer combination employed by das Neves and co-authors [29] to sequence the ITS region of *Cereus peruvianus* remains unspecified. The blue line on the tree highlights the Hylocereinae sequences determined in our study. The green line highlights sequences obtained by other authors. The dark red line highlights the ITS sequences of both Hylocereinae and Echinocereinae processed by Plume et al. [14] using the ITSC1-F + ITSC1-R primers. Brackets on the right delineate major lineages: ECHI—Echinocereinae; CERE—Cereinae; TRICH—Trichocereinae; CACT—Cacteae; OPUN—Opuntieae. LYM—Lymanbensonieae. Subtribes are defined according to the classification of Nyffeler and Eggli [1] and de Vos et al. [7]. For a comprehensive breakdown of depositors and their respective primers, see Table 1.

The distinctiveness of the pseudogenes amplified and sequenced using the ITSC1-F/ITSC1-R primers is also evident in phylogenetic reconstructions. As shown in Figure 7, all sequences are distributed among three major clades. Two of these clades (the “red group”) comprise a strongly supported subclade (BS = 97) containing species of the subtribe Hylocereinae and a second subclade (BS = 87) containing species of the subtribe Echinocactinae. All sequences belonging to the “red group” were generated by Plume et al. [14].

The third clade, designated the “green group” (BS = 99), includes both the Hylocereinae and Echinocactinae species sequenced in the present study (highlighted by a blue outline) and all other cactus species from various subtribes and tribes retrieved from GenBank. Notably, three sequences of *Pachycereus pringlei* are present in the dataset. Two of them (JX289549 and JX289550) belong to the “red group”, whereas sequence AY181584, sequenced by Arias et al. [21], is assigned to the “green group”. It is evident that all sequences comprising the “green group” were generated using primer pairs different from ITSC1-F and ITSC1-R (see Figure 7 legend and Table 1).

### 2.2. Phylogenetic Relationships of Selenicereus and Related Taxa Inferred from ITS Sequences

**Figure 8:**
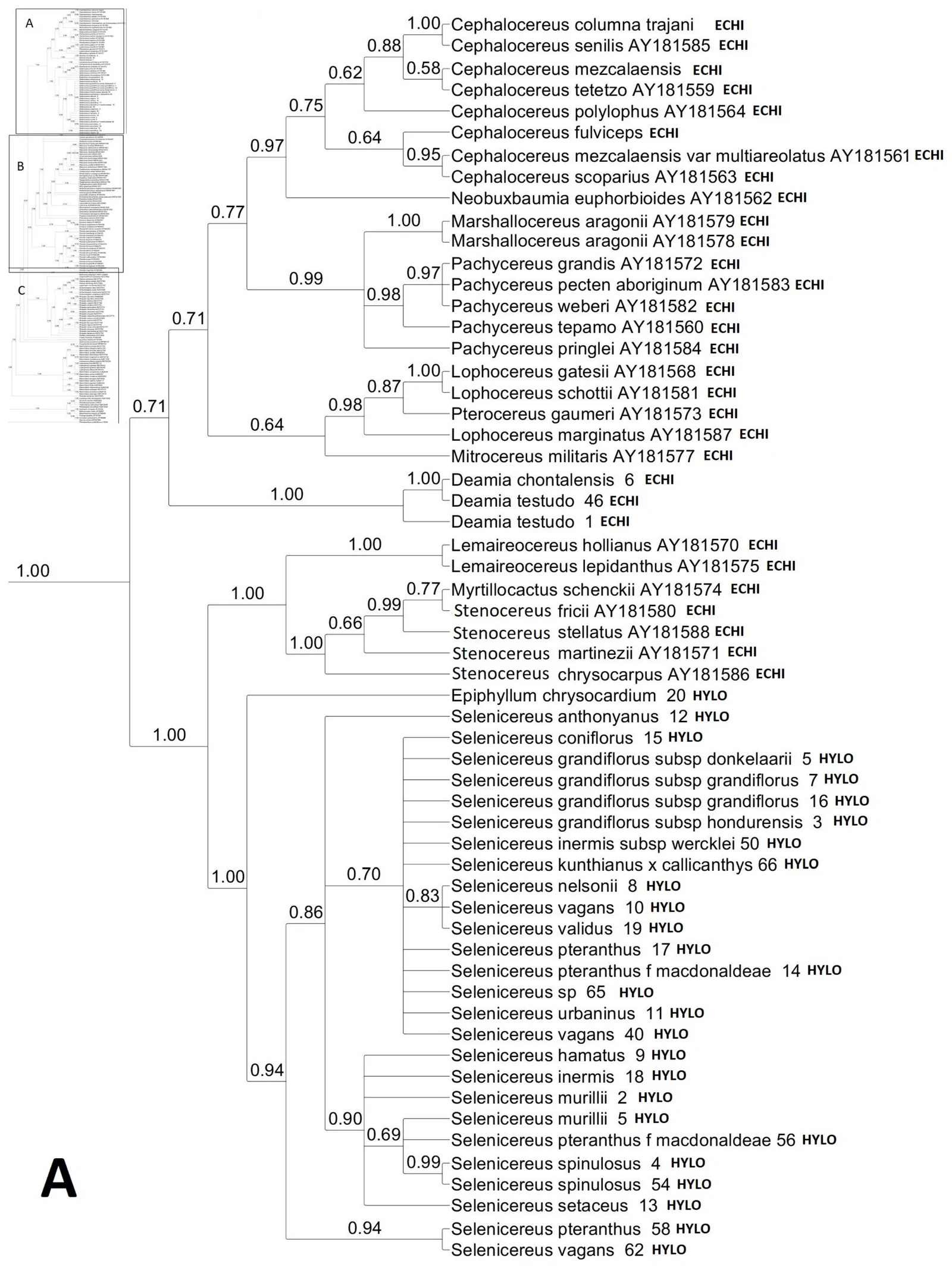

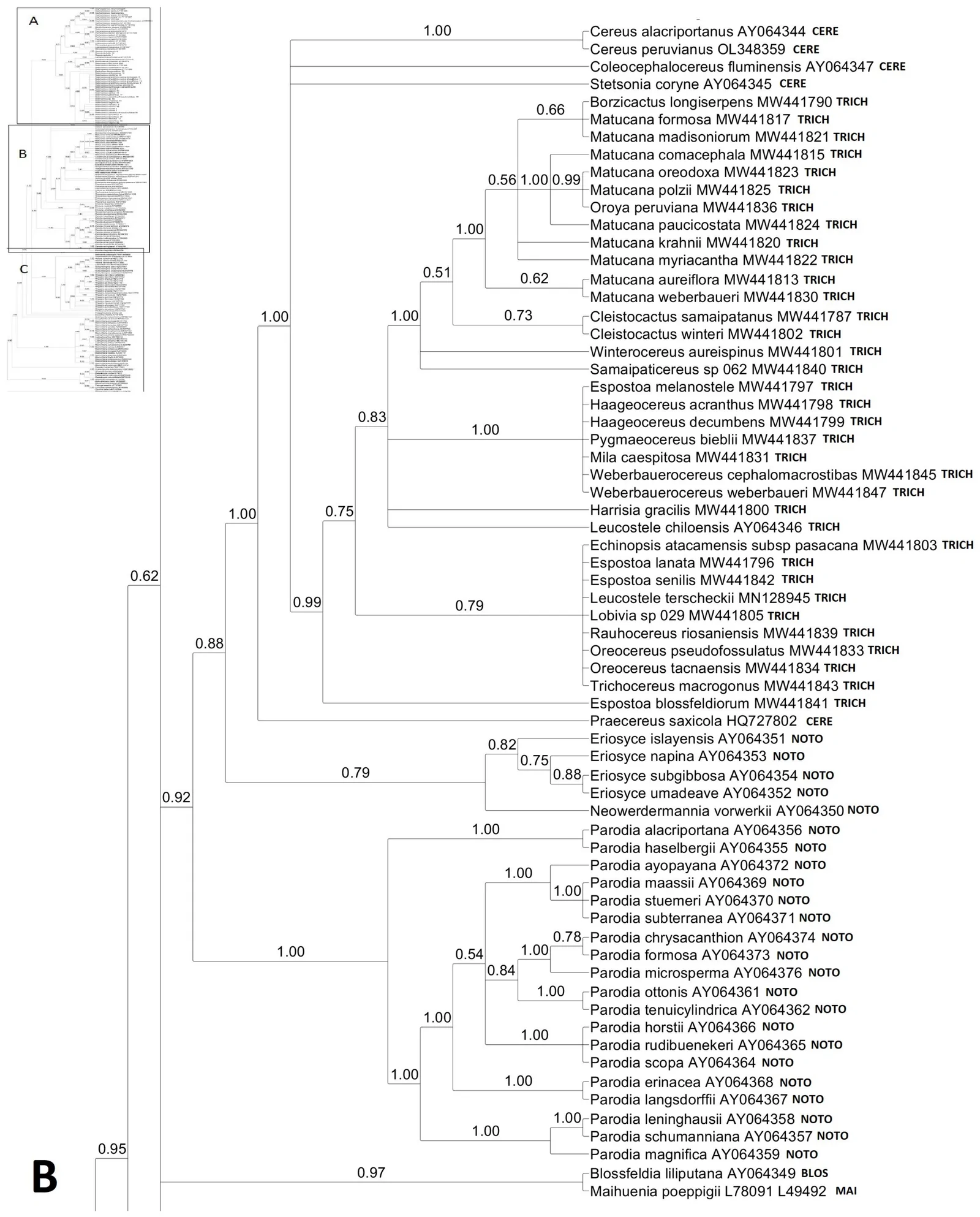

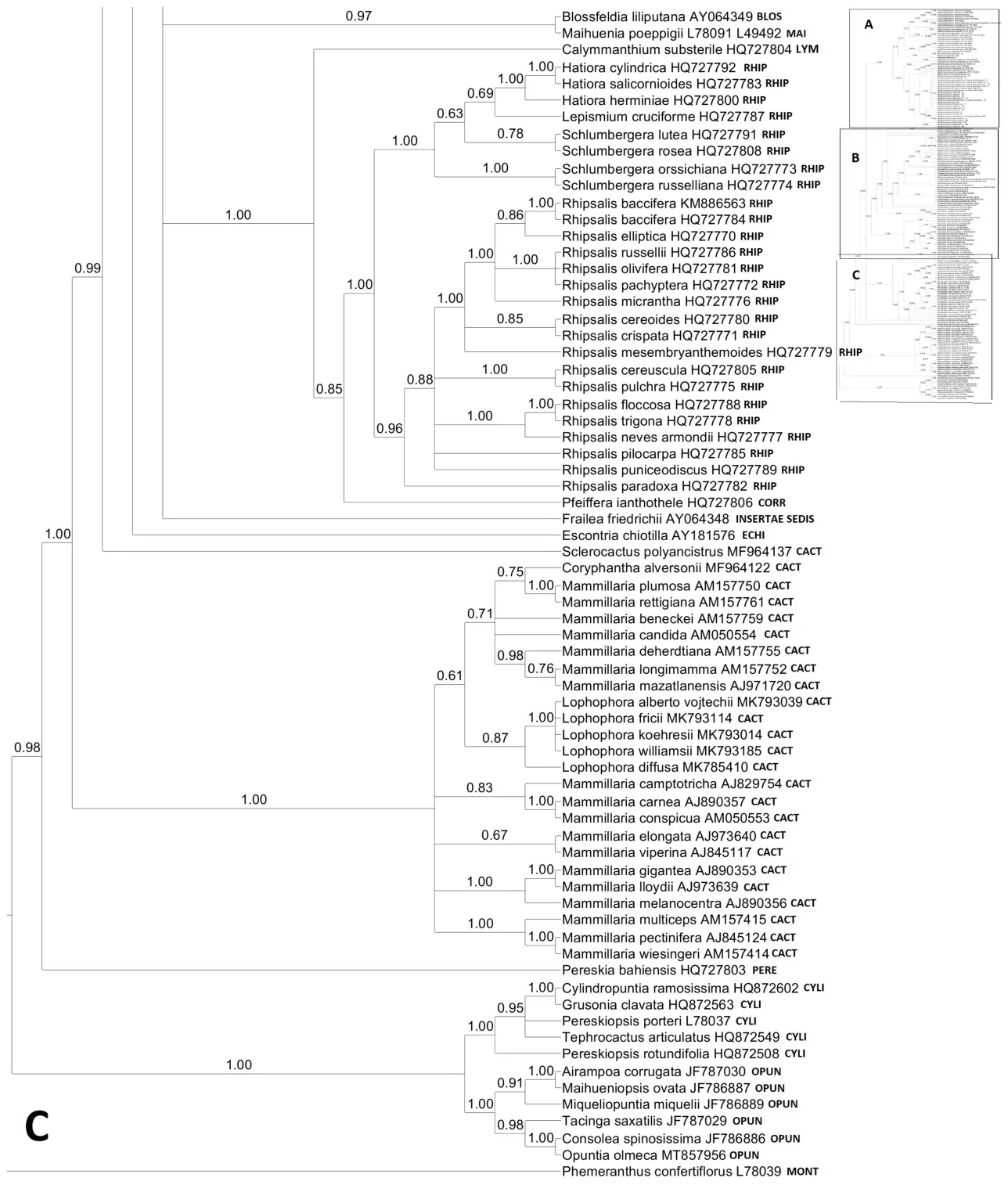
(**A**–**C**). Phylogenetic tree of the subfamily Cactoideae inferred from nuclear ribosomal ITS sequences using Bayesian inference (see also Supplementary Materials, Figure S1). Node values represent Bayesian posterior probabilities (PP). Tribes and subtribes are designated according to de Vos et al. [7]: Pereskioideae (PERE); Opuntioideae-Cylindropuntieae (CYLI); Cactoideae-Blossfeldieae (BLOS); Cactoideae-Cacteae (CACT); Cactoideae Lymanbensonieae (LYM); Cactoideae-Phyllocacteae-Corryocactinae (CORR); Cactoideae-Phyllocacteae-Hylocereinae (HYLO); Cactoideae-Phyllocacteae-Echinocereinae (ECHI); Cactoideae-Rhipsalideae (RHIP); Cactoideae-Notocacteae (NOTO); Cactoideae-Cereeae-Rebutiinae (REBU); Cactoideae-Cereeae-Cereinae (CERE); Cactoideae-Cereeae-Trichocereinae (TRICH); and the outgroup family Montiaceae (MONT)). As the phylogenetic tree presented in Figure 8 is rather large, it has been divided into three panels (**A**–**C**) to facilitate visualization.

The phylogenetic tree reconstructed using Bayesian inference and based on ITS1–5.8S rDNA–ITS2 sequences generated in different laboratories with various primer pairs, excluding ITSC1-F and ITSC1-R, is shown in Figure 8 and Figure S1. In this tree, all *Selenicereus* species form a single clade with a posterior probability (PP) of 0.94, while *Epiphyllum chrysocardium* constitutes their sister lineage (Figure 8A and Figure S1). Both genera belong to the subtribe Hylocereinae, which was recovered as monophyletic in the present study with maximal support (PP = 1.00).

The sister clade to Hylocereinae comprises four species of *Stenocereus*, one species of *Myrtillocactus*, and two species of *Lemaireocereus* (Figure 8A and Figure S1). All remaining species and genera assigned to the subtribe Echinocereinae form a separate clade with relatively low support (PP = 0.71). Thus, Echinocereinae is recovered as paraphyletic in our analysis (Figure 8A and Figure S1).

Species of *Deamia* are grouped together with representatives of Echinocereinae in the ITS1–5.8S rDNA–ITS2 tree.

## 3. Discussion

### 3.1. Cactaceae Phylogenetic Tree Based on ITS1–5.8S rDNA–ITS2 Sequences

The topology of the Cactaceae phylogeny inferred from the multicopy nuclear ITS1–5.8S rDNA–ITS2 sequence is largely congruent with phylogenetic hypotheses based on hundreds of low-copy nuclear genes [7]. In general, the same overall topology has also been recovered using chloroplast markers [15,45].

In the ITS-based tree, the subtribe Hylocereinae is recovered as a strongly supported monophyletic group (PP = 1.00). Its sister lineage consists of species belonging to the subtribe Echinocereinae. The genus *Deamia* is grouped together with Echinocereinae species, in agreement with phylogenies based on chloroplast markers [15,45] and low-copy nuclear genes [7].

The sister group of Phyllocacteae comprises the clades (Cereeae + Notocacteae; PP = 0.92), (Rhipsalideae + *Calymmanthium* [incertae sedis]; PP = 1.00), and (Blossfeldieae + *Maihuenia*; PP = 0.97), together with the isolated species/genus *Faihuenia friedrichii* (Figure 8B,C and Figure S1). According to POWO (https://powo.science.kew.org/) the last name is a synonym of *Frailea schilinzkkyana* (F.Haage ex K.Schum.) Britton & Rose. Outside this superclade lie the isolated species *Escontria chiotilla* and *Sclerocactus polyancistrus* (Engelm. & J.M.Bigelow) Britton & Rose.

Particular attention should be paid to the isolated position of *Calymmanthium substerile* F. Ritter in the ITS phylogeny. This monotypic genus, characterized by a highly restricted distribution and a unique floral morphology [1], shows no close affinity to *Leptocereus*, contrary to the hypothesis proposed by Buxbaum [46]. Its phylogenetic position may therefore justify recognition of a separate tribe, Calymmantheae, as proposed by Anderson [47] and Arias et al. [12].

The type species of the genus *Pfeiffera*, *P. ianthothele* (Monv. ex Salm-Dyck) F.A.C.Weber, occupies an unusual phylogenetic position. Based on morphology, the genus was originally assigned to the subtribe Rhipsalidinae of the former tribe Hylocereeae [48]. Subsequent chloroplast DNA analyses demonstrated that *P. ianthothele* does not belong to the tribe Rhipsalideae [37,49]. It is currently placed within the subtribe Corryocactinae of the tribe Phyllocacteae [1,7]. Nevertheless, our ITS-based phylogenetic reconstructions place *P. ianthothele* at a peripheral position within Rhipsalideae, albeit with moderate support (PP = 0.86). This discrepancy may be consistent with a history of reticulate evolution or ancient hybridization in the genus *Pfeiffera*, although additional evidence from nuclear and plastid genomes will be required to test this hypothesis.

Phylogenetic trees inferred from both chloroplast markers [1,37] and low-copy nuclear genes [7] consistently place *Escontria chiotilla* within Echinocereinae. The position of this species in our ITS phylogeny may therefore be artefactual. Arias et al. [21] demonstrated that the ITS sequence of *E. chiotilla* differs markedly from those of other Phyllocacteae–Echinocereinae taxa. Additional investigation of rDNA evolution in the genus *Escontria* is therefore warranted.

A well-supported clade corresponding to the subtribes Cereinae and Trichocereinae within the tribe Cereeae sensu lato Nyffeler and Eggli [1] is recovered in our phylogeny with bootstrap support of 99 and posterior probability PP = 1.00.

Within our sampling, the subtribe Rebutiinae is represented only by *Stetsonia coryne* (C.F.Först.) Britton & Rose, which occupies an isolated position within the Cereeae clade. This placement is consistent with the conclusions of Romeiro-Brito et al. [50] and de Vos et al. [7], who argued that *Stetsonia* should be excluded from the subtribe Rebutiinae.

In the ITS-based phylogeny, species assigned to the tribe Notocacteae form a distinct clade (PP = 0.92), with the exception of *Frailea*, which has traditionally been included in Notocacteae on morphological grounds [8]. A similar pattern is observed in chloroplast-based phylogenies, where *Frailea* also fails to cluster within any recognized notocactoid lineage [13,37,51]. Consequently, de Vos et al. [7] recognized *Frailea* as the sole representative of a separate monogeneric tribe, Fraileeae.

Phylogenetic trees inferred from both chloroplast markers [1,37] and low-copy nuclear genes [7] indicate that the genus *Escontria* belongs to the subtribe Echinocereinae. Consequently, the topological placement of this lineage on our ITS-based tree might be erroneous. The fact that the ITS region of this genus exhibits significant divergence from other members of the Phyllocacteae-Echinocereinae lineage was previously demonstrated by Arias et al. [21]. This biological discrepancy warrants further systematic investigation of rDNA evolution within the genus *Escontria*.

At the periphery of the phylogenetic tree are two deeply divergent monophyletic lineages, Cylindropuntieae (PP = 1.00) and Opuntioideae-Opuntieae (PP = 1.00) (Figure 8C and Figure S1), both belonging to the monophyletic subfamily Opuntioideae (PP = 1.00). Also recovered as monophyletic are the tribe Cacteae (PP = 1.00) and one of the earliest-diverging representatives of the family Cactaceae, *Pereskia* [52].

Finally, our ITS-based phylogeny confirms the monophyly of the tribe Cacteae and of the two tribes recognized within Opuntioideae, Cylindropuntieae and Opuntieae.

### 3.2. ITS1–5.8S rDNA–ITS2 as a DNA Barcode for Hylocereinae

The internal transcribed spacers ITS1 and ITS2 are variable, often species-specific DNA markers in plant genomes. B Both the entire ITS1–5.8S rDNA-ITS2 region and its components (ITS1 or ITS2) serve as the most widely used DNA barcodes for angiosperms [17,18].

Although each haploid plant genome contains hundreds to thousands of 35S rRNA gene copies [53,54,55,56], these genes are generally assumed to be homogenized, meaning that most copies are highly similar or identical in sequence [57,58,59]. However, notable exceptions exist. Relatively high ITS variability has been reported in *Cycas* [60], *Asclepias* [61], and ×*Elyhordeum* [62]. Moreover, several species of Cactaceae have been shown to possess an unexpectedly high degree of rDNA polymorphism [22,63].

In the context of the present study, it is important to note that Plume et al. [14] developed the novel primer pair ITSC1-F and ITSC1-R after detecting length polymorphisms in ITS1–5.8S rDNA–ITS2 amplicons generated with the ITS4 and ITS5A primers. The new primer pair enabled the amplification of more uniform-length but highly specific ITS sequences from several species of the tribe Phyllocacteae.

In contrast, all other researchers who sequenced ITS regions in Cactaceae employed a wide variety of primer combinations, including the commonly used ITS1/ITS4 and ITS5/ITS4 pairs, as well as several unique and less conventional primer sets (see Table 1). Apparently, the use of different primer pairs had little influence on the sequencing results. It is highly likely that, regardless of the primer combinations employed, all laboratories amplified and sequenced the same rDNA paralogs, presumably the predominant rDNA paralogs present in cactus genomes. As a consequence, ITS sequences generated by different research groups using different primer pairs were largely congruent with one another, yet consistently differed from the ITS1–5.8S–ITS2 sequences obtained by Plume et al. [14] (Figure 2 and Figure 3).

In contrast, our sequences differed from the 5.8S sequences of *Selenicereus* previously deposited by Plume et al. [14] by 23–24 SNPs (p-distance = 0.14–0.16). This level of divergence is comparable to that observed between our sequences and the 5.8S sequence of *Amborella trichopoda* Baill. LC669891 [64], which differed by 25 SNPs, and is even greater than the divergence between our sequences and the 5.8S rDNA of the Nymphaeaceae (*Victoria amazonica* (Poepp.) Klotzsch [65], which differed by 16 SNPs, or the 5.8S rDNA of *Arabidopsis thaliana* (L.) Heynh. [66], which differed by only 7 SNPs.

The most likely explanation is that the primer-binding sites targeted by the primers designed by Plume et al. [14] are specific to an orphan copy of a 45S rRNA pseudogene, a conclusion that is fully supported by our bioinformatic analyses. Indeed, the ITSC1-F/ITSC1-R primer pair exhibits high affinity for a single-copy rDNA pseudogene located on chromosome 9 of *Selenicereus* and is unable to anneal the canonical rDNA arrays located within the NOR.

On the other hand, the fact that the ITS variants reported by Plume et al. [14] can be detected not only in species of the subtribe Hylocereinae but also in two species of Echinocereinae (Figure 3) is consistent with our observation that 5.8S rDNA-P-related sequences occur both in *Selenicereus monacanthus* (CM104742.1, chromosome 9) and in the genome of *Carnegiea gigantea* (JAKOGI010000397.1). This suggests that the highly degraded ribosomal repeat carrying this pseudogenic variant and translocated to another chromosome was already present in one or several copies in the genome of the common ancestor of the tribe Phyllocacteae, no later than the early Miocene [2,67,68].

## 4. Materials and Methods

### 4.1. Plant Material and DNA Extraction

For this study, the nuclear ITS1–5.8S rDNA–ITS2 region sequences were determined for 16 species and subspecies of the genus *Selenicereus*, two species of *Deamia*, and *Epiphyllum chrysocardium*. Plant tissues were sampled from the living greenhouse collections of the Peter the Great Botanical Garden of the Komarov Botanical Institute of the Russian Academy of Sciences, St. Petersburg, Russia (hereafter abbreviated as BIN); the N.V. Tsitsin Main Botanical Garden of the Russian Academy of Sciences, Moscow (hereafter Tsitsin BG); and the Moscow State University Botanical Garden “Apothecary Garden”, Moscow (hereafter MGU). The examined species, sample code, and their collection number/Genbank accession number are as follows:

*Deamia chontalensis* (Alexander) Doweld: samples #6 (BIN 10879/PV902793) and S60 (MGU 17041001/PV902794);

*Deamia testudo* (Karw. ex Zucc.) Britton & Rose: samples #1 (BIN 244925/PX457882), #46 (Tsitsin BG 0000.03910/PX457883), and #64 (MGU 17041004/PX457884);

*Epiphyllum chrysocardium* Alexander: sample #20 (BIN 244951/PV902806);

*Selenicereus anthonyanus* (Alexander) D.R. Hunt: samples #12 (BIN 244969/PX446996) and #63 (MGU 17129002/PX457870);

*Selenicereus grandiflorus* (L.) Britton & Rose ssp. *grandiflorus*: samples #7 (BIN 8626/PX447019), #15 (BIN 8648/PV902814), #16 (BIN 244884/PX447018);

*Selenicereus grandiflorus* ssp. *donkelaarii* (Salm-Dyck) Ralf Bauer: sample #5 (BIN 8637/PV915527);

*Selenicereus grandiflorus* ssp. *hondurensis* (K. Schum. ex Weing.) Ralf Bauer: samples #3 (BIN 7141/PX447032), #52 (MGU 171290162B/PX447033), and #53 (MGU 171290162A/PX447034);

*Selenicereus hamatus* (Scheidw.) Britton & Rose: samples #9 (BIN 13349/PV915533) #41 (Tsitsin BG 0000.03904/PV902602);

*Selenicereus inermis* (Otto ex Pfeiff.) Britton & Rose: sample #18 (BIN 263408/PX452308);

*Selenicereus murrillii* Britton & Rose: samples #2 (BIN 251668/PV625054) and #55 (MGU 17129020/PV625055);

*Selenicereus nelsonii* (Weing.) Britton & Rose: sample #8 (BIN 241128/PX457769);

*Selenicereus pteranthus* (Link ex A. Dietr.) Britton & Rose: samples #17 (BIN 7427/PV608660) and #58 (Tsitsin BG 17129022/PV608661);

*Selenicereus pteranthus* f. *macdonaldieae* (Hook.) Ralf Bauer: samples #14 (BIN 241876/PX457767) and #56 (MGU 171290221/PX457766);

*Selenicereus setaceus* (Salm-Dyck ex DC.) Werderm.: sample #13 (BIN 16793/PX457784);

*Selenicereus spinulosus* (DC.) Britton & Rose: samples #4 (BIN 12329d/PX457858) and #54 (MGU 17129026/PX457859);

*Selenicereus urbanianus* (Gürke & Weing.) Britton & Rose: sample #11 (BIN 8495/PX457861);

*Selenicereus vagans* (K. Brandegee) Britton & Rose: samples #10 (BIN 94233/PV608662), #40 (Tsitsin BG 0000.03911/PV608663), and #62 (MGU 17129033/PV608664);

*Selenicereus validus* S. Arias & U. Guzmán: sample #19 (BIN 8657/PX457866);

*Selenicereus wercklei* (F.A.C. Weber) Britton & Rose: sample #50 (MGU 17129015B/PX561141);

*Selenicereus* sp.: samples #65 (MGU 17129000A/PX457885) and #66 (MGU 17129021x/PX586169).

Total genomic DNA was isolated from fresh tissue using a modified cetyltrimethylammonium bromide (CTAB) protocol described by Doyle and Doyle [69].

### 4.2. PCR Amplification and Sequencing

The nuclear ribosomal ITS1–5.8S rDNA-ITS2 region was amplified via polymerase chain reaction (PCR) using the forward primer ITS1P (5′–AACCTTATCATTTAGAGGAAGG–3′) [31] and the reverse primer ITS4 (5′–TCCTCCGCTTATTGATATGC–3′) [32]. Amplification was conducted under the following cycling conditions: an initial denaturation step at 95 °C for 5 min; followed by 30 cycles of denaturation at 94 °C for 1 min, annealing at 52 °C for 1 min, and extension at 72 °C for 1 min; with a final elongation step at 72 °C for 10 min.

The resulting PCR products were purified and sequenced in both directions using the BigDye Terminator v3.1 Cycle Sequencing Kit (Applied Biosystems, Foster City, CA, USA) according to the manufacturer’s protocol. Capillary electrophoresis was performed on an ABI 3500 sequencer (Thermo Fisher Scientific, 168 Third Avenue, Waltham, MA USA) at the Shared Research Facility “Cell and Molecular Technologies in Plant Science” of the Komarov Botanical Institute, Russian Academy of Sciences.

### 4.3. Ribotype Network and Phylogenetic Analyses

The 5.8S rDNA ribotype network was constructed using the statistical parsimony algorithm implemented in TCS software (Brigham Young University, Provo, UT, USA) [34]. The resulting network graphics were visualized using the tcsBU program (University of Porto, Porto, Portugal) developed by Múrias dos Santos et al. [35].

Comparative sequence analysis and alignme nt of the newly generated ITS1–5.8S rDNA-ITS2 nucleotide data were performed using MEGA12 (Temple University, Philadelphia, PA, USA) [70]. Phylogenetic relationships among the obtained sequences were first evaluated using the Minimum Evolution (ME) method [71]. Evolutionary distances were computed using the Kimura 2-parameter (K2P) model [72]. The ME tree space was explored via the Close-Neighbor-Interchange (CNI) algorithm at a search level of 1, with the initial tree generated using the Neighbor-Joining algorithm [73]. This dataset encompassed 60 nucleotide sequences. All ambiguous positions were addressed using the pairwise deletion option, yielding a final dataset of 637 positions. Nodal support was assessed by bootstrap analysis with 1000 pseudoreplicates [74].

To resolve the broader phylogenetic placement of *Selenicereus*, *Epiphyllum*, and *Deamia* within the family Cactaceae, a Bayesian inference (BI) approach was employed [75]. The optimal nucleotide substitution model was determined using jModelTest v.2.1.10 (Department of Biochemistry, Genetics and Immunology, University of Vigo, Vigo, Spain) [76]. Indel regions were coded as binary characters using SeqState v.1.4.1 [77] and appended to the primary alignment. Bayesian analysis was conducted using MrBayes v.3.2.2 under a GTR+I+G substitution model. The Markov chain Monte Carlo (MCMC) analysis was run for 1 million generations, with sampling every 100 generations; the initial 25% of the sampled trees were discarded as burn-in to estimate posterior probabilities (PP). Maximum Likelihood analysis was performed using IQ-TREE v.2.3.6 (Computational Molecular Evolution Group, Center for Integrative Bioinformatics Vienna (CIBIV), University of Vienna, Vienna, Austria) [78] under the same GTR+I+G model, with branch support assessed using the ultrafast bootstrap option with 1000 replicates.

### 4.4. Genomic Data Mining and BLAST Searches

To evaluate the prevalence and chromosomal localization of the newly identified functional ribotypes alongside the pseudogenic variants published by Plume et al. [14], available reference genome assemblies representing 12 species of Cactaceae were retrieved from the NCBI database: *Selenicereus monacanthus* (GCA_047496255.1), *Selenicereus undatus* (GCA_017589665.1), *Cereus jamacaru* (GCA_051401755.1), *Cereus fernambucensis* (GCA_024363205.1), *Opuntia basilaris* (GCA_043229145.1), *Lophophora williamsii* (GCA_964133935.1), *Carnegiea gigantea* (GCA_029747015.1), *Pachycereus pringlei* (GCA_002740445.2), *Lophocereus schottii* (Engelm.) Britton & Rose (GCA_002740545.2), *Pereskia humboldtii* Britton & Rose (syn. of *Pereskia horrida* DC.) (GCA_002740485.2), *Stenocereus thurberi* (Engelm.) Buxb. (GCA_002740465.2), *Eriosyce duripulpa* (F.Ritter) P.C.Guerrero & Helmut Walter (GCA_051621435.1). Using the local BLAST+ (MEGA12) suite, the assemblies were searched for conserved 5.8S rDNA fragments corresponding to the functional ribotype identified in the present study (designated as 5.8S rDNA-A) and to the putative pseudogenic ribotype reported by Plume et al. [14] (designated as 5.8S rDNA-P).

## 5. Conclusions

The ITS1–5.8S rDNA–ITS2 region has long been recognized as a powerful tool for plant species identification in DNA barcoding studies and as a widely used nuclear marker in phylogenetic reconstructions [17,18,19,79]. In the present study, we demonstrate that numerous *Selenicereus* sensu lato sequences deposited in GenBank, together with sequences of *Pachycereus pringlei* (JX289549, JX289550) and *Stenocereus griseus* (JX289547, JX289548) generated using the ITSC1-F/ITSC1-R primer pair, most likely represent rare pseudogenic copies of the 35S rRNA gene repeat. In our phylogenetic analyses, these sequences form a distinct clade that is strongly isolated from all other representatives of the Cactaceae. The pseudogenic nature of these sequences likely explains why previous attempts to use ITS sequences as DNA barcodes for species identification in *Selenicereus* were considered unsuccessful by Huy et al. [80].

In contrast, the ITS1–5.8S rDNA–ITS2 sequences obtained in the present study exhibit close phylogenetic affinities to ITS sequences from representatives of other cactus subtribes and tribes. Several lines of evidence indicate that these sequences represent functional rDNA copies: they constitute the predominant form of rDNA in the analyzed genomes, occur within canonical NOR-associated repeat arrays, and retain all three conserved motifs within the 5.8S rDNA region that are commonly used to distinguish functional copies from pseudogenic variants.

The phylogenetic relationships among Hylocereinae and related lineages inferred from these sequences are highly congruent with phylogenies previously reconstructed using chloroplast markers [12,13,14,15], complete plastome sequences [16], and approximately 300 low-copy nuclear loci [7]. In particular, phylogenetic analyses based on the ITS sequences generated in this study, together with ITS sequences retrieved from GenBank, support the monophyly of Hylocereinae, Rhipsalideae, and Trichocereinae, as well as the tribes Phyllocacteae (with the exception of *Escontria* and *Pfeiffera*), Rhipsalideae, Notocacteae, and Cylindropuntieae.

Taken together, our results demonstrate that the ITS1–5.8S rDNA–ITS2 region of functional 35S rRNA genes represents a reliable and informative DNA barcode for *Selenicereus* sensu lato and provides a robust marker for phylogenetic studies within the Cactaceae.

## Figures and Tables

**Table 1:** Oligonucleotide primers utilized for the amplification and sequencing of the nuclear ribosomal ITS1–5.8S rDNA-ITS2 region in Cactaceae.

Papers	Primers	Primer Forward	Primer Reverse	Authors of Primers
Plume et al. 2013 [14]	C1FR	ITSC1F GTTTCCGTAGGTGAACTTGTGG	ITSC1R GTGCTTTCAAAACCTTAGTGCT	Plume et al. 2013 [14]
Majeský, L. direct submission	W14	ITS1 TCCGTAGGTGAACCTGCGG	ITS4 TCCTCCGCTTATTGATATGC	White et al. 1990 [32]
Quipildor et al. 2018 [36]	W14	ITS1 TCCGTAGGTGAACCTGCGG	ITS4 TCCTCCGCTTATTGATATGC	White et al. 1990 [32]
This paper	W1P4	ITS1P AACCTTATCATTTAGAGGAAGG	ITS4 TCCTCCGCTTATTGATATGC	Ridgway et al. 2003 [31]; White et al. 1990 [32]
Arias et al. 2003 [21]	W54	ITS5 GGAAGTAAAAGTCGTAACAAGG	ITS4 TCCTCCGCTTATTGATATGC	White et al. 1990 [32]
Harpke and Peterson 2006 [22]	W54	ITS5	ITS4 TCCTCCGCTTATTGATATGC	White et al. 1990 [32]
Majure et al. 2012 [25]	W54	ITS5	ITS4 TCCTCCGCTTATTGATATGC	White et al. 1990 [32]
Thornhill et al. 2017 [27]	W5A4	ITS5A CCTTATCATTTAGAGGAAGGAG	ITS4 TCCTCCGCTTATTGATATGC	Stanford et al. 2000 [38]; White et al. 1990 [32]
Hershkovitz, M.A. and Zimmer, E.A. 1997 [20]	H	18NRBlr TTCCGTAGGTGAACCTGCG	26A TTTCTTTTCCTCCGCT	Hershkovitz and Zimmer 1997 [20]: Keith Humby et al. 1988 [39]
Calvente et al. 2011 [23]	S	17SE ACGAATTCATGGTCCGGTGAAGTGTTCG	26SE TAGAATTCCCCGGTTCGCTCGCCGTTAC	Sun et al. 1994 [40]
Yesson et al. 2011 [24]	B54	5F GGAAGTAAAAGTCGTAACAAGG.	4R TCCTCCGCTTATTGATATGC	Baldwin, 1993 [41]
Yesson et al. 2011 [24]	YA	ITS-AF GTCGTAACAAGGTTTCCATA	ITS-AR CTTAAACTCAGCGGGCAGCC	Yesson et al. 2011 [24]
Santos et al. 2013 [43]		ITS92 AAGGTTTCCGTAGGTGAAC	ITS4 TCCTCCGCTTATTGATATGC	Oger-Desfeux et al. 1996 [44]; White et al. 1990 [32]
Schaefer, H., Weigelt, B. and Hu, Y. (direct submission)	B12	(1) CCTGCCCTTTGTACACACC	(2) TCTCGGCAACGGATATCTCG	von Balthazar et al. (2000) [42]

## Data Availability

All data generated or analyzed during this study are contained within the article.

## Supplementary Materials

The following supporting information can be downloaded at: https://www.mdpi.com/article/10.3390/ijms27167166/s1.
