# Supplementary material for: Nuclear Ribosomal ITS1–5.8S rDNA-ITS2 Region as a Phylogenetic Marker for Selenicereus grandiflorus and Related Species (Cactaceae: Cactoideae: Phyllocacteae)
Source: Int J Mol Sci. 2026 Aug 11;27(16):7166. doi: 10.3390/ijms27167166 (PMC13513733; doi:10.3390/ijms27167166)
Supplement: Supplementary file 1 [file ijms-27-07166-s001.zip › Table S1 Supplemen1.pdf]

Table S1 Supplement

List of sequences of the ITS1-5.8S rDNA-ITS2 region of species from different genera of Cactaceae, used by us to construct the phylogenetic trees (Figure 7 & 8) and the 5.8S rDNA ribotype system (Figure 1).

Species	accession number	Reference
**subfamily Blossfeldioideae (or subfamily Cactoideae, tribe Blossfeldieae)**		
Blossfeldia liliputana Werderm.	AY064349	Nyffeler and Eggli, 2010
**subfamily Cactoideae, tribe Cacteae, subtribe Cactinae**		
Coryphantha alversonii (J.M. Coult.) Orcutt, syn. of *Pelecyphora alversoni* (J.M.Coult.) D.Aquino & Dan.Sanchez	MF964122	Thornhill et al., 2017
Lophophora koehresii (Řiha) Bohata, Myšák & Šnicer, syn. of *Lophophora diffusa* (Croizat) Bravo	MN158150	Majesky,L. 2019; Direct submission
Mammillaria beneckei C.Ehrenb., syn. of *Oehmea beneckei* (C.Ehrenb.) Buxb.	AM157759	Harpke and Peterson, 2006
**Subfamily Cactoideae, tribe Cacteae, subtribe Echinocactinae**		
Echinocactus grusonii Hildm., syn. of *Kroenleinia grusonii* (Hildm.) Lodé	MK660269	Jha, V.J. et al., 2019; Direct submission
Sclerocactus polyancistrus (Engelm. & J.M.Bigelow) Britton & Rose	MF964137	Thornhill et al., 2017
**Subfamily Cactoideae, tribe Cereeae, subtribe Cereinae**		
Cereus peruvianus C.F.Först., syn. of *Stenocereus griseus (*Haw.) Buxb.	OL348364	Neves, A.F. et al. 2021; Direct submission
Pilosocereus pachycladus F.Ritter	OP188781	Romeiro-Brito,M., et al., 2022; direct submission
Praecereus saxicola (Morong) N.P. Taylor, syn. of *Monvillea saxicola* (Morong) A.Berger	HQ727802	Calvente et al., 2011
Stetsonia coryne (C.F.Först.) Britton & Rose	AY064345	Nyffeler and Eggli, 2010
Xiquexique gounellei (F.A.C.Weber ex K.Schum.) Lavor & Calvente	OP188778	Romeiro-Brito,M. et al., 2022; Direct submission
**Subfamily Cactoideae, tribe Cereeae, subtribe Rebutiinae**		
Uebelmannia pectinifera Buining	OP188777	Romeiro-Brito,M., et al., 2022; Direct submission
**Subfamily Cactoideae, tribe Cereeae, subtribe Trichocereinae**		
Borzicactus longiserpens (Leuenb.) G.J.Charles	MW441790	Schlumpberger, B. et al., 2021; Direct submission
Cleistocactus winteri D.R. Hunt *subsp. colademononis* (Diers et Krahn) D.R. Hunt	MW441802	Schlumpberger, B. et al., 2021; Direct submission
Cremnocereus albipilosus M.Lowry & Winberg	MW441795	Schlumpberger, B. et al., 2021; Direct submission
Espostoa lanata (Kunth) Britton & Rose	MW441796	Schlumpberger, B. et al., 2021; Direct submission
Haageocereus decumbens (Vaupel) Backeb.	MW441799	Schlumpberger, B. et al., 2021; Direct submission
Harrisia gracilis (Mill.) Britton	MW441800	Schlumpberger, B. et al., 2021; Direct submission
Leucostele chiloensis (Colla) Schlumpb.	AY064346	Nyffeler and Eggli, 2010

Leucostele terscheckii (J.Parm. ex Pfeiff.) Schlumpb.	MN128945	Quiroga et al., 2019
Lobivia sp. Britton & Rose, syn. of *Echinopsis* Zucc.	MW441805	Schlumpberger, B. et al., 2021; Direct submission
Matucana oreodoxa (F.Ritter) Slaba	MW441823	Schlumpberger, B. et al., 2021; Direct submission
Mila caespitosa Britton & Rose	MW441831	Schlumpberger, B. et al., 2021; Direct submission
Oreocereus tacnaensis F.Ritter	MW441834	Schlumpberger, B. et al., 2021; Direct submission
Oroya peruviana (K.Schum.) Britton & Rose, syn. of *Matucana peruviana* (K.Schum.) G.J.Charles	MW441836	Schlumpberger, B. et al., 2021; Direct submission
Pygmaeocereus bieblii Diers	MW441837	Schlumpberger, B. et al., 2021; Direct submission
Rauhocereus riosaniensis Backeb.	MW441839	Schlumpberger, B. et al., 2021; Direct submission
Samaipaticereus sp. 062 Cárdenas	MW441840	Schlumpberger, B. et al., 2021; Direct submission
Trichocereus macrogonus var. *pachanoi* (Britton & Rose) Albesiano & R.Kiesling, syn. of Echinopsis pachanoi (Britton & Rose) H.Friedrich & G.D.Rowley	MW441843	Schlumpberger, B. et al., 2021; Direct submission
Weberbauerocereus cephalomacrostibas (Werderm. & Backeb.) F.Ritter	MW441848	Schlumpberger, B. et al., 2021; Direct submission
Yungasocereus inquisivensis (Cárdenas) F.Ritter ex Eggli	MW441849	Schlumpberger, B. et al., 2021; Direct submission
**subfamily Cactoideae, tribe Phyllocacteae, subtribe Corryocactinae**		
Pfeiffera ianthothele (Monv. ex Salm-Dyck) F.A.C.Weber	HQ727806	Calvente et al., 2011
**Subfamily Cactoideae, tribe Phyllocacteae, subtribe Echinocereinae**		
Carnegiea gigantea (Engelm.) Britton & Rose	AY181566	Arias et al., 2003
Cephalocereus senilis (Haw.) K.Schum.	AY181585	Arias et al., 2003
Cephalocereus columna-trajani (Karw. ex Pfeiff.) K.Schum.	AY181565	Arias et al., 2003
Escontria chiotilla (F.A.C.Weber ex K.Schum.) Rose	AY181576	Arias et al., 2003
Marshallocereus aragonii (F.A.C.Weber) Backeb.	AY181579	Arias et al., 2003
Myrtillocactus schenckii (J.A.Purpus) Britton & Rose	AY181574	Arias et al., 2003
Pachycereus grandis Rose	AY181572	Arias et al., 2003
Pachycereus pringlei (S.Watson) Britton & Rose	AY181584	Arias et al., 2003
Pachycereus pringlei (S.Watson) Britton & Rose	JX289549-JX289550	Plume et al., 2013
Stenocereus griseus (Haw.) Buxb.	JX289547-JX289548	Plume et al., 2013
Stenocereus fricii Sánchez-Mej.	AY181580	Arias et al., 2003
Stenocereus martinezii (J.G.Ortega) Buxb.	AY181571	Arias et al., 2003

Stenocereus stellatus (Pfeiff.) Riccob.	AY181588	Arias et al., 2003
**subfamily Cactoideae, tribe Phyllocacteae, subtribe Hylocereinae**		
Selenicereus atropilosus Kimnach	JX289551-JX289556	Plume et al., 2013
Selenicereus grandiflorus (L.) Britton & Rose subsp. grandiflorus	JX289773-JX289795	Plume et al., 2013
Selenicereus grandifloras (L.) Britton & Rose *subsp. donckelaari* (Salm-Dyck) Ralf Bauer	JX289765-JX289771,	Plume et al., 2013
Selenicereus hamatus (Scheidw.) Britton & Rose	JX289762	Plume et al., 2013
Selenicereus inermis (Otto) Britton & Rose	JX289581-JX289587	Plume et al., 2013
Selenicereus ×innesii Kimnach, syn. of *×Aporberocereus innesii* (Kimnach) Doweld	JX289557-JX289570	Plume et al., 2013
Selenicereus pteranthus (Link ex A.Dietr.) Britton & Rose	JX289763-JX289764, JX289772, JX289788, JX289796-JX289799	Plume et al., 2013
Selenicereus spinulosus (DC.) Britton & Rose	JX289572-JX289575	Plume et al., 2013
Selenicereus vagans (K.Brandegee) Britton & Rose	JX289800-JX289802	Plume et al., 2013
Selenicereus sp. OP-2013	JX289752, JX289571, JX289576-JX289580	Plume et al., 2013
**Subfamily Cactoideae, tribe Fraileeae**		
Frailea friedrichii Buining & G.Moser, syn. of *Frailea* schilinzkyana (F.Haage ex K.Schum.) Britton & Rose subsp. *schilinzkiana* (*Frailea schilinzkyana* (F. Haage ex K. Schum.) Britton et Rose)	AY064348	Nyffeler and Eggli, 2010
**subfamily Cactoideae, tribe Lymanbensonieae**		
Calymmanthium substerile F.Ritter	HQ727804	Calvente et al., 2011
**subfamily Cactoideae, tribe Notocacteae**		
Eriosyce napina (Phil.) Katt.	AY064353	Nyffeler and Eggli, 2010
Neowerdermannia vorwerkii Frič	AY064350	Nyffeler and Eggli, 2010
Parodia langsdorfii (Lehm.) D.R.Hunt	AY064367	Nyffeler and Eggli, 2010
**subfamily Cactoideae, tribe Rhipsalideae**		
Hatiora herminiae (Porto & A.Cast.) Backeb. ex Barthlott	HQ727800	Calvente et al., 2011
Lepismium cruciforme (Vell.) Miq.	HQ727787	Calvente et al., 2011
Rhipsalis baccifera (J.S.Muel.) Stearn	KM886563	Ornelas,J.F. and Rodriguez-Gomez,F., 2019; Direct submission

Schlumbergera rosea (Lagerh.) Calvente & Zappi, syn. of *Rhipsalidopsis rosea* (Lagerh.) Britton & Rose	HQ727808	Calvente et al., 2011
Subfamily **Maihuenioideae**		
Maihuenia poeppigii (Otto et Pfeiff.) F.A.C.Weber ex K.Schum.	L78091	Hershkovitz and Zimmer, 1997
**Subfamily Opuntioideae, tribe Cylindropuntieae**		
Cylindropuntia ramosissima (Engelm.) F.M.Knuth	HQ872602	Yesson et al., 2011
Grusonia clavata (Engelm.) H. Rob.	HQ872563	Yesson et al., 2011
Pereskiopsis porteri (Brandegee ex F.A.C.Weber) Britton & Rose	L78037	Hershkovitz and Zimmer, 1997
Pereskiopsis rotundifolia (DC.) Britton & Rose	HQ872508	Yesson et al., 2011
**Subfamily Opuntioideae, tribe Opuntieae**		
Consolea spinosissima (Mill.) Lem.	JF786886	Majure et al., 2012
Miqueliopuntia miquelii (Monv.) F.Ritter	JF786889	Majure et al., 2012
Opuntia olmeca Joel Pérez, J. Reyes et Brachet, syn. of *Opuntia tehuacana* S.Arias & U.Guzmán	MT857956	Martinez-Gonzalez, C.R., 2021; Direct submission
Tacinga saxatilis (F.Ritter) N.P.Taylor& Stuppy	JF787029	Majure et al., 2012
Tunilla corrugata (Salm-Dyck) D.R.Hunt & Iliff, syn. of *Airampoa corrugata* (Salm-Dyck) Doweld	JF787030	Majure et al., 2012
**subfamily Opuntioideae, tribe Pterocacteae**		
Maihueniopsis ovata (Pfeiff.) F. Ritter	JF786887	Majure et al., 2012
Tephrocactus articulatus (Pfeiff.) Backeb.	HQ872549	Yesson et al., 2011
Family **Montiaceae**		
Phemeranthus confertiflorus (Greene) Hershk., syn. of *Phemeranthus parviflorus* (Nutt.) Kiger	L78039	Hershkovitz and Zimmer, 1997
Family **Montiaceae**		
Pereskia bahiensis Gürke	HQ727803	Calvente et al., 2011

Synonyms in Table S1 Supplement were verified using the POWO Kew website https://powo.science.kew.org/
